# Functional Properties of Polyurethane Ureteral Stents with PLGA and Papaverine Hydrochloride Coating

**DOI:** 10.3390/ijms22147705

**Published:** 2021-07-19

**Authors:** Magdalena Antonowicz, Janusz Szewczenko, Joanna Jaworska, Katarzyna Jelonek, Kamil Joszko, Bożena Gzik-Zroska, Paweł M. Nuckowski, Piotr Bryniarski, Zbigniew Paszenda, Damian S. Nakonieczny, Karla Čech Barabaszová, Janusz Kasperczyk

**Affiliations:** 1 Department of Biomaterials and Medical Devices Engineering, Faculty of Biomedical Engineering, Silesian University of Technology, Roosevelta 40 St., 41-800 Zabrze, Poland; Janusz.Szewczenko@polsl.pl (J.S.); Bozena.Gzik-Zroska@polsl.pl (B.G.-Z.); Zbigniew.Paszenda@polsl.pl (Z.P.); Damian.Nakonieczny@polsl.pl (D.S.N.); 2 Centre of Polymer and Carbon Materials of the Polish Academy of Sciences, 34 Marii Skłodowskiej-Curie St., 41-819 Zabrze, Poland; jjaworska@cmpw-pan.edu.pl (J.J.); kjelonek@cmpw-pan.edu.pl (K.J.); jkasperczyk@cmpw-pan.edu.pl (J.K.); 3 Department of Biomechatronics, Faculty of Biomedical Engineering, Silesian University of Technology, Roosevelta 40 St., 41-800 Zabrze, Poland; Kamil.Joszko@polsl.pl; 4 Materials Research Laboratory, Faculty of Mechanical Engineering, Silesian University of Technology, 18A Konarskiego St., 44-100 Gliwice, Poland; Pawel.Nuckowski@polsl.pl; 5 Department of Urology, School of Medicine with the Division of Dentistry in Zabrze, Medical University of Silesia, 13-15 3 Maja St., 41-800 Zabrze, Poland; piotr.bryniarski@hotmail.com; 6 Nanotechnology Centre, CEET, VŠB-Technical University of Ostrava, 17. Listopadu 15/2172, 708 00 Ostrava-Poruba, Czech Republic; karla.cech.barabaszova@vsb.cz

**Keywords:** ureteral stent, PLGA with papaverine hydrochloride coatings, retention strength, break strength, dynamic frictional force, radiopacity

## Abstract

Despite the obvious benefits of using ureteral stents to drain the ureters, there is also a risk of complications from 80–90%. The presence of a foreign body in the human body causes disturbances in its proper functioning. It can lead to biofilm formation on the stent surface, which may favor the development of urinary tract infections or the formation of encrustation, as well as stent fragmentation, complicating its subsequent removal. In this work, the effect of the polymeric coating containing the active substance-papaverine hydrochloride on the functional properties of ureteral stents significant for clinical practice were assessed. Methods: The most commonly clinically used polyurethane ureteral Double-J stent was selected for the study. Using the dip-coating method, the surface of the stent was coated with a poly(D,L-lactide-glycolide) (PLGA) coating containing the papaverine hydrochloride (PAP). In particular, strength properties, retention strength of the stent ends, dynamic frictional force, and the fluoroscopic visibility of the stent during X-ray imaging were determined. Results: The analysis of the test results indicates the usefulness of a biodegradable polymer coating containing the active substance for the modification of the surface of polyurethane ureteral stents. The stents coated with PLGA+PAP coating compared to polyurethane stents are characterized by more favorable strength properties, the smaller value of the dynamic frictional force, without reducing the fluoroscopic visibility.

## 1. Introduction

Ureteral stents used for treatment of the upper urinary tract are implantable devices that support the flow of the urine from the kidneys to the bladder. Ureteral stents can be used short-term for up to 30 days, or long-term for several months or even years depending on the cause and course of treatment. Despite over a century of research and clinical experience supported by advances in materials and biomedical engineering, no ideal ureteral stent has yet been developed. The ideal ureteral stent should be characterized by [1,2,3,4]:

- Ease of implantation and removal,
- Position stability after implantation,
- Good visibility during imaging,
- Good biocompatibility, and biodegradable after the treatment process is completed,
- Resistance to incrustation.

Moreover, it should [4,5,6,7]:

- Ensure continuity of urine flow,
- Not cause reflux,
- Not cause peri-implant reactions in the patient,
- Not cause irritation.

The most commonly used stent construction today is “Double-J” DJ. It was first introduced by Finney in 1978. The term Double-J refers to the shape of the stent ends, the purpose of which is to anchor the stent in the kidney and in the bladder in order to prevent its displacement [8]. The DJ stent is placed in the ureter, which functions as a low pressure, stretchy tube with its own peristalsis. Ureters are made up of smooth muscle fibers. The wall of the ureter consists of fibrous coat, the muscular coat, and the mucosa. Due to contractions of the muscle membrane, peristaltic movements of the ureter take place, which transport urine from the renal pelvis to the bladder. During contraction, the mucosa of the ureter forms longitudinal folds, which cause obstruction of the ureter, which is an indispensable condition for effective peristatic movement. The entire ureter carries out coordinated peristaltic movements, and the autonomic nervous system, in particular the sympathetic nervous system, is responsible for indirect control over the mechanism of creating and spreading the peristaltic wave. The pressure generated by the peristalsis of the ureter can be as high as 1898 cm H_2_O. Systolic waves disappear when the bladder is too full, but intensify when the lumen of the ureter is closed, e.g., by a stone. This type of contraction is very painful and is known as renal colic. The ureters are strongly innervated by the senses, hence there is severe pain in the event of problems with the outflow of urine [9,10,11]. Therefore, understanding the complexity of the ureter along with its peristalsis is important in carrying out the in vitro studies. The mechanical behavior of ureteral tissue can be predicted by modeling the peristaltic flow. The authors [12] concluded that the ureteral tissue is stiffer in the longitudinal direction than in the circumferential direction and that perhaps the collagen fibers are along the axial axes.

**Figure 1:**
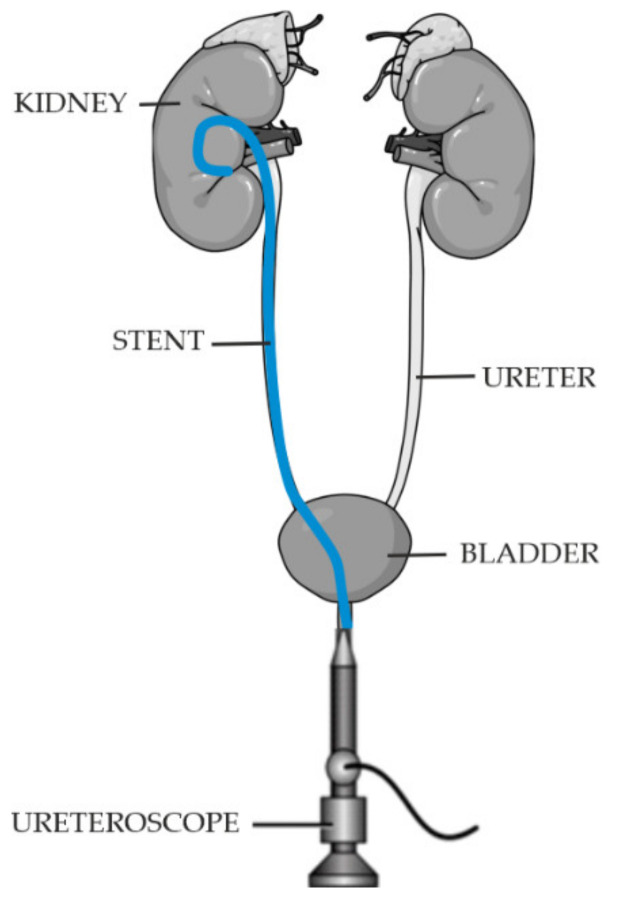
Ureteral stent placement.

The most commonly used technique of ureteral stent implantation is the transurethral method, performed endoscopically [13]. Ureteral stents are introduced into the ureter through the cystoscope working canal under fluoroscopic control (Figure 1).

However, complications are also associated with the presence of a ureteral stent in the ureters. After the implantation of stents, the patient often experiences discomfort, which negatively affects the quality of life, leading, among others, to local inflammation, causing hematuria or irritating urination [14]. A common management strategy in this case is pharmacological treatment with the use of antispasmodic drugs, causing relaxation of the ureter. Among others, alpha 1-blockers and antimuscarinic drugs are used [15]. Moreover, one of the drugs used is papaverine hydrochloride (C_20_H_21_NO_4_·HCI), which has relaxation properties. The papaverine hydrochloride has been shown to be more effective in the treatment of mild colic pain in patients with refractory pain to treatment than conventional medications [16,17]. However, oral administration of drugs is associated with numerous undesirable effects and a limited effectiveness of local exposure [18,19]. This resulted in the initiation of works aimed at alternative drug delivery to the treatment area. 

In addition, during ureteral stent implantation, damage of the stent by overstraining or breaking is possible, which can lead to complete rupture of the stent in the body [20,21]. This has serious consequences for the patient as fragments of the fracture stent may migrate into the bladder and, worse, towards the renal pelvis [22,23]. The authors [24,25,26] showed fragmentation of ureteral stents which required medical intervention by percutaneous nephroscopy and surgery. Therefore, in addition to the ability of ureteral stents to prevent microbial growth causing incrustation, they should also have appropriate mechanical properties to prevent fracture during *in vivo* use, while alleviating pain experienced by the patient.

In order to improve the mechanical properties [27,28], biodegradable coatings on ureteral stents are being developed. However, the literature data presents only partial results which do not determine the performance of ureteral stents [29,30,31,32].

PLGA is the most frequently used biodegradable polymer in various applications like tissue engineering [33,34], healing bone defects [35,36], and in a controlled release of encapsulated drugs [37]. PLGA is FDA-approved material for medical purposes and its widespread use results from its known biocompatibility [37]. PLGA is also suitable for biodegradable coatings/layers and can be easily processed. Cauda et al. developed a heparin-loaded PLGA coating on Double-J polyurethane stent in order to reduce incrustation [38]. However, PLGA was also successfully used as a coating on other substrates, e.g., on Ti–6Al–4V alloy in the form of a gentamicin-loaded layer intended for prosthetic hips [39]. PLGA used in our study was synthesized with Zr(acac)_4_ initiator (contrary to Sn(oct)_2_ in commercially available bioresorbable polyesters) and has been previously presented as a suitable polymer coating for metallic implants [39,40,41,42,43] or as a coating on Parylene C [44]. The possibility of encapsulating drugs within the layers provides additional advantages like local delivery of the drug, which makes it even more attractive. Therefore, we chose this material for its drug-loaded bioresorbable layer ensuring local release. Our study attempts to determine the effect of biodegradable PLGA coating with an active substance-papaverine hydrochloride applied to polyurethane stents on the improvement of functional properties. By developing drug-eluting coatings on ureteral stents, additional functionality was obtained.

This can contribute to an increase in the effectiveness of treatment, reduce the number of complications during implantation and improve the quality of patients’ lives.

## 2. Materials and Methods

### 2.1. Modification of Ureteral Stents

**Figure 2:**
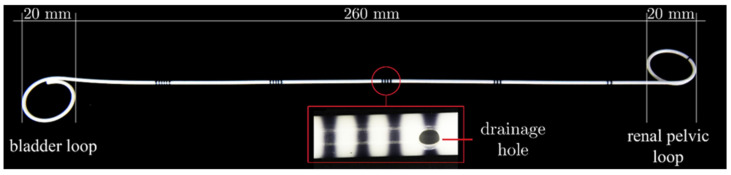
Polyurethane Double-J ureteral stents.

Polyurethane Double-J ureteral stents (S) with an external diameter of 4.8 Fr for clinical use Figure 2, and polyurethane ureteral stents with PLGA and papaverine hydrochloride coating (SP), were selected for research. A copolymer of lactide and glycolide with the participation of 85:15 comonomer units (D,L-LL/GG), with an amorphous structure and molar mass Mn = 74 kDa, Mw = 195 kDa was used as the coating. The copolymer synthesis was carried out in two stages [45]. The copolymers were synthesized in bulk by the ring opening polymerization (ROP) of glycolide (Purac) and D,L lactide (Purac) at 130 °C for 24 h, then at 120 °C for 48 h under argon using the initiator: zirconium acetylacetonate (Zr(acac)_4_). In the next step, the synthesized PLGA polymer was dissolved in dichloromethane CH_2_Cl_2_ to give a 1% solution. The active substance was added to the resulting solution the diastolic drug papaverine hydrochloride (C_20_H_21_NO_4_·HCl) (Sigma-Aldrich, Darmstadt, Germany) (20% of the drug by weight of the polymer) (PLGA+PAP).

### 2.2. Modification of Ureteral Stents

Surface modification of polyurethane stents was made by dip coating methods using a dip coater (MTI Corporation, Richmond, CA, USA). One cycle of dipping, 30 s immersion was used. During immersion, the stent was on the guide. In order to ensure even coverage of the stent surface with a polymer coating, after immersion the stent was placed in a horizontal position in a self-constructed device. It provided axial rotation of the stent placed on the Teflon guide and was equipped with speed regulation. The stents were then dried for two hours at room temperature at 12 rpm. Subsequently, they were kept in a vacuum dryer (50,000−8000 Pa) at 21 °C for 7 days.

### 2.3. Stent Pretreatment Procedures In Vitro

Prior to testing the mechanical properties of polyurethane stents (S) and coated polyurethane stents (SP), they were placed in artificial urine for the required period of time at 37 ± 1 °C. Artificial urine was prepared based on previously reported concentrations [46]. Reagents were used to prepare the AU solution from Acros Organics (New Jersey, USA) and demineralized water with a conductivity of 0.06 μS/cm. Artificial urine content was 6.17 g/dm^3^ NaCl, 4.59 g/dm^3^ NaH_2_PO_4_, 0.944 g/dm^3^ Na_3_C_6_H_5_O_7_, 0.463 g/dm^3^ MgSO_4_, 2.408 g/dm^3^ Na_2_SO_4_, 4.75 KCl g/dm^3^, 0.638 g/dm^3^ CaCl_2_ and 0.043 g/dm^3^ Na_2_C_2_O_4_. The pH of the artificial urine solution was 5.5 ± 0.2.

### 2.4. Mechanical Properties of Ureteral Stents

#### 2.4.1. Break Strength

The static tensile test was performed in accordance with the recommendations of the standard: ASTM F 1828-97 [47] and PN-EN ISO 527:2012 [48] with a tensile speed of 20 mm/min, using the MTS Insight 2 testing machine (MTS Systems, Eden Prairie, MN, USA) with a 100 N force sensor and MTS TestSuite software. The distance between the grippers was 2.54 cm. The stent samples in the gripper were positioned with the drainage hole in the center. On the basis of the tests, the Young’s modulus E [MPa], the maximum breaking force F_max_ [N], tensile strength R_m_ [MPa] and elongation at break A [%] were determined. A static tensile test was performed on polyurethane (S) and coated stents (SP) both before and after soaking in artificial urine for 30 days. Five studies were performed for each type of stent.

#### 2.4.2. Retention Strength

**Figure 3:**
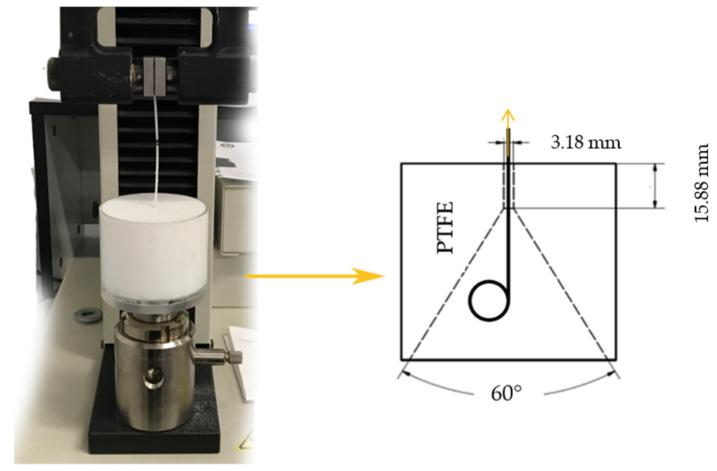
Stand for testing the retention strength of the distal and proximal ureteral stents ends.

The retention strength test of the proximal and distal ends of the ureteral stents were tested using the MTS Insight 2 testing machine (MTS Systems, Eden Prairie, MN, USA) with a 100 N force sensor and the MTS TestSuite Software in accordance with the recommendations of ASTM F 1828-97 [47]. A Teflon funnel block was prepared for the tests, through which the stent ends were drawn through the opening Figure 3. Before the test, the funnel and the tested stent were immersed (for 1 min in order to achieve thermal equilibrium) in artificial urine at a temperature of 37 ± 1 °C. Then, one of the stent ends was placed with the help of a guide in the centered opening of the funnel, while the other end (allowing for shape change) was placed in the handle of the testing machine. The procedure for withdrawing the tip from the funnel was performed at a speed of 20 mm/min. The retention strength tests of ureteral stents ends were carried out for polyurethane stents (S), coated stents (SP) and for stents soaked in artificial urine for 30 days at 37 ± 1 °C. Five ends of each type of stent were used for the study.

#### 2.4.3. Dynamic Frictional Force

**Figure 4:**
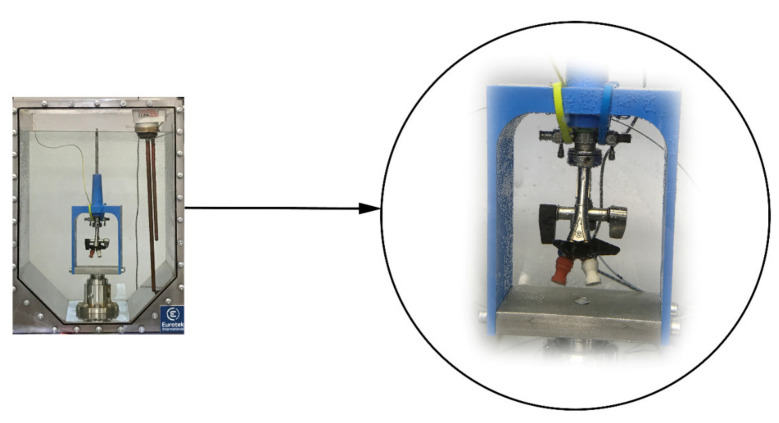
Test apparatus for dynamic frictional force test.

Measurement of the dynamic friction force acting on the outer surface of the stent, in order to simulate the passage of the stent through the endoscope during implantation, was carried out on the MTS criterion 43 testing machine (MTS Systems, Eden Prairie, MN, USA) with a 100 N force sensor and the MTS TestSuite software in accordance with the recommendations of the ASTM F 1828-97 standard [47]. Before starting the test, a guide was inserted into the stent, then it was placed in a cystoscope and the whole in demineralized water at a temperature of 37 ± 1 °C Figure 4. The other tip was placed in the gripper of the machine, which was pulled out at a constant speed of 500 mm/min. During the measurement, the value of the dynamic friction force was determined. Polyurethane (S) and coated stents (SP) were tested, with five sampled of each type.

#### 2.4.4. Radiopacity

Qualitative X-ray phase analysis was performed in the X’Pert PRO MPD X-ray diffractometer (PANalytical, Almelo, the Netherlands), equipped with a cobalt lamp (λKαCo = 1.79 Å) and a PIXcel 3D detector on the diffracted beam axis. Measurements were made in the angular range from 40° to 120° 2Θ with a 0.05° step and time per step equal to 100 s. The results were processed using the High Score Plus software (v. 3.0e, Panalytical, Almelo, The Netherlands) with the PAN-ICSD inorganic crystal structure database. Samples taken from the polyurethane stent and the coated stent were tested.

#### 2.4.5. Statistical Analysis

In the study, for the mechanical research method, the sample populations were presented as means with standard deviation. In order to determine the significance of differences for *p <* 0.05, the one-way and two-way analysis of variance (ANOVA) for the obtained results were used.

## 3. Results

### 3.1. Break Strength

**Figure 5:**
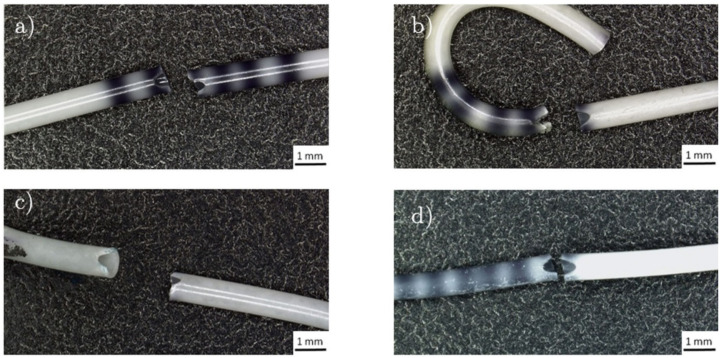
Ureteral stents after a static tensile test: (**a**) polyurethane stent before soaking, (**b**) stent with a PLGA+PAP coating before soaking, (**c**) polyurethane stent after soaking, (**d**) stent with a PLGA+PAP coating after soaking.

The parameter values describing the mechanical properties of dry and wet ureteral stents, determined on the basis of a static tensile test, are summarized in Table 1. Regardless of the type of the tested stent, its rupture took place in the area of the drainage hole Figure 5. Based on the analysis of the results, different values of mechanical properties were found for stents in the initial state and with the PLGA+PAP coating applied. Moreover, the effect of soaked stents was significant (*p* < 0.05) for the value of mechanical properties for the polyurethane stents and coated stents. Coated stents were characterized by higher strength properties compared to stents in their initial state, regardless of whether they were dry or soaked. Moreover, dry stents were characterized by more than twice the value of Young’s modulus (*p* < 0.05). Twice as much elongation at break was observed for soaked stents with a polymer coating than for dry stents (*p* < 0.05).

### 3.2. Retention Strength Test

The analysis of the results of the retention strength test of the proximal and distal ends of stents in Table 2 showed different mean values of the maximum force depending on the type of the tested stent. Stents with PLGA+PAP coating were characterized by a lower retention force value of the distal and proximal ends compared to stents in the initial state, both in a dry and soaked state. Moreover, the coating applied to ureteral stents did significantly change the recorded value of the maximum retention force (*p* < 0.05), both in the dry and soaked states. In contrast, the tips of the dry and soaked stents, both coated and uncoated, showed a significant increase in force (*p <* 0.05).

### 3.3. Dynamic Frictional Force

The values of the average dynamic friction force for polyurethane stents in the initial state was 0.281 (16) N, and with the PLGA+PAP coating, was 0.063 (43) N (results notation (mean (standard deviation)) in accordance with [49]). The application of the coating significantly changed the registered values of the dynamic friction force (*p* < 0.05). The application of the PLGA+PAP coating to the polyurethane stent caused a more than fourfold decrease in the value of the average dynamic friction force.

### 3.4. Radiopacity

**Figure 6:**
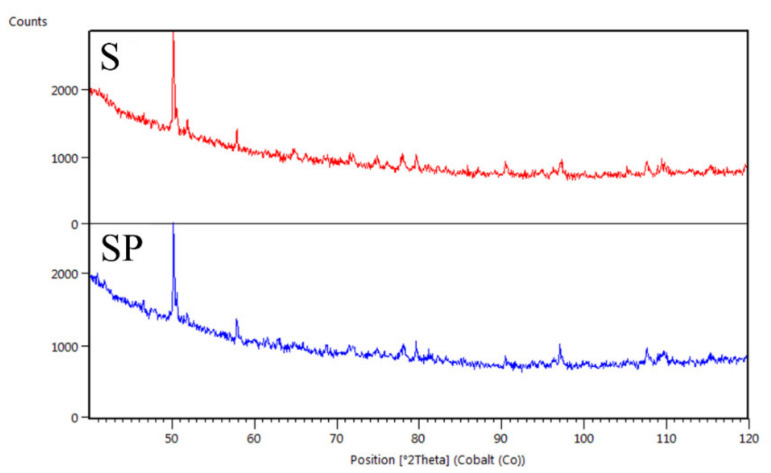
XRD patterns of the polyurethane stent (S) and the coated stent (SP).

**Figure 7:**
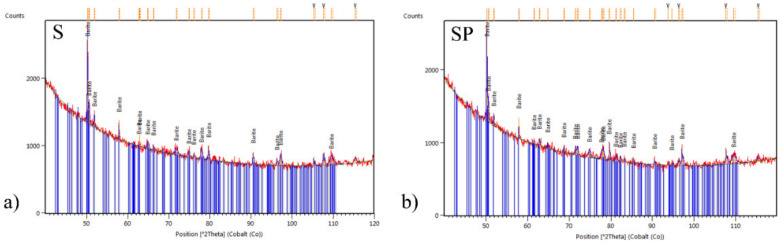
XRD patterns of the barium sulphate (BaSO_4_) standard for the ureteral stents: (**a**) polyurethane (S), (**b**) polyurethane with PLGA+PAP coating (SP).

Qualitative X-ray phase analysis provided the basis for the identification of phases occurring in the tested materials. On all recorded diffractograms, irrespective of the tested stent (Figure 6 and Figure 7), the background elevation in the range of low angles, characteristic for the amorphous structure, was observed. Diffraction lines in angular positions corresponding to the crystalline (orthorhombic primitive) structure of barium sulphate BaSO_4_—barite (according to the characteristics card-PAN-ICSD:98-003-3730) were also identified [50]. The tests performed showed that the crystalline barium sulfide is characterized by a much higher X-ray absorption coefficient compared to the amorphous polymer substrate. On this basis, it can be concluded that in the studied ureteral stents, it mainly serves as a contrast. Based on the analysis of the intensity of the diffraction lines recorded for barium sulphate, it can be concluded that the applied polymer coating is characterized by an amorphous structure and does not reduce the visibility of the stent in the X-ray image.

## 4. Discussion

In spite of improvements in ureteral stents, none of the biomaterial used for stent development has perfect mechanical properties and biocompatibility. Apart from infection and incrustation problems connected with using ureteral stents is their fragmentation in the ureter. This is mainly related to the design of the stent. Tensile strength and stent end retention strength are properties of a stent that can have an overall effect on performance and comfort. Transverse drainage holes in the structure of a ureteral stent significantly reduce their strength, and the greater their number, the greater the velocity of urine that flows through the lumen of the stent, which is beneficial for the treatment of ureteral strictures [51,52,53,54,55,56]. This was reflected in the standards for stent strength tests. It is recommended that the stent rupture should be in the area of the drainage hole [47]. Based on the obtained results, it was found that regardless of the type of stent tested, its rupture occurred in the area of the drainage hole. The stents with the PLGA+PAP coating proposed by the authors of the study were characterized by higher strength properties than polyurethane stents, both in dry and soaked states. The obtained results for polyurethane stents were similar to the results obtained by other authors [53,57]. The more favorable strength properties of stents with PLGA+PAP coating, compared to polyurethane stents, indicate that its surface functionalization with a biodegradable polymer improves its functional properties. The study of retention strength of stent ends, according to the recommendations of the standard [47], showed that the application of the PLGA+PAP coating resulted in a reduction of the retention strength of both the distal and proximal stent ends compared to the ends of polyurethane stents, both in dry and soaked states. This is a disadvantageous phenomenon that can lead to migration of the stent, e.g., by displacement of the stent into the kidney or the bladder, causing irritation that is very burdensome for the patient and can lead to inflammation. Literature data indicate that the onset of inflammation is also associated with an incorrect technique of stent implantation through damage to the urinary tract epithelium [58,59]. Therefore, a very important parameter is the value of the friction force between them during implantation. This value cannot be determined in vitro. However, in the first stage of implantation, the stent is moped through the cystoscope. The surface of the stent displaced in the cystoscopy and interacting with its walls may be damaged, which during the implantation stage in the ureter may increase the friction force and, consequently, damage the ureter epithelium. In order to determine the interaction of the ureteral stent surface with the cystoscope, tests of the dynamic friction force between the stent surface and the inside of the cystoscope lumen are performed. Based on the analysis of the obtained results of dynamic friction force tests between the stent surface and the inside of the cystoscope canal, it was found that the applied PLGA+PAP coating caused a fourfold reduction in the value of the dynamic friction force compared to the uncoated stent. This clearly indicates the beneficial effect of the PLGA+PAP coating on the analyzed functional features of ureteral stents. In this case, the migration of the stent in the ureter is less likely to cause irritation and, consequently, inflammation, since the dynamic friction force resulting from the application of this coating is four times lower. Visibility measurements in fluoroscopy were also carried out to determine the usefulness of the applied modification of the ureteral stent surface. On the basis of the obtained results, it was found that the coating does not reduce the fluoroscopic visibility of the stent.

Summing up, it can be stated that undoubtedly, ureteral stents with the proposed PLGA+PAP coating have good utility properties and, very significantly, do not deteriorate fluoroscopic visibility. This form of ureteral stents allows for precise positioning of their position, which in turn improves the effectiveness and safety of the procedure.

The next stage of the research will be to determine the effect of the PLGA+PAP coating applied to polyurethane stents on the antibacterial properties and cytotoxicity. As part of this, research on cell cultures and then animal models will be performed. 

## 5. Conclusions

Based on the research, the following generalizations were formulated. The PLGA+PAP polymer coating applied to the surface of polyurethane stents contribute to:

- Increase in its strength properties, in dry and soaked condition,
- Quadruple reduction of dynamic friction force,
- lowering the value of the retention strength of the stent tips: proximal and distal, in a dry and soaked state.

Moreover, it does not deteriorate the fluoroscopic visibility.

## Figures and Tables

**Table 1:** Mechanical properties of ureteral stents results notation (mean (standard deviation)) in accordance with [49].

	DRY		SOAKED	
**Mechanical Properties**	**Polyurethane Stents**	**Stents with PLGA+PAP Coating**	**Polyurethane Stents**	**Stents with PLGA+PAP Coating**
Young’s Modulus E [MPa]	20.4(52)	23.1(21)	8.86(33)	9.2(11)
max. Breaking Force F_max_ [N]	29.40(60)	38.0(25)	32.2(49)	35.6(62)
Tensile strength R_m_ [MPa]	22.10(50)	28.7(19)	24.3(37)	26.9(47)
Elongation at break A [%]	143(35)	153(28)	303(36)	293(47)

**Table 2:** Retention force values of ureteral stent ends results notation (mean (standard deviation)) in accordance with [49].

SAMPLE	DRY				SOAKED			
	**Polyurethane** **Stents**		**Stents with PLGA+PAP Coating**		**Polyurethane Stents**		**Stents with PLGA+PAP Coating**	
**End**	proximal	distal	proximal	distal	proximal	distal	proximal	distal
**Fmax [N]**	0.41 (13)	0.38 (16)	0.1370 (40)	0.1290 (80)	1.64 (18)	1.742 (40)	1.400 (46)	1.354 (49)

## Data Availability

Data are contained within the article.
